# Reducing the Carbon Footprint in Maternal Healthcare

**DOI:** 10.7759/cureus.90596

**Published:** 2025-08-20

**Authors:** Kalpana Singh, Renin Peter AA Raj, Treesa Thomas, Abdulqadir J Nashwan

**Affiliations:** 1 Nursing &amp; Midwifery Research Department, Hamad Medical Corporation, Doha, QAT; 2 Medicine, Aston Medical School, Birmingham, Birmingham, GBR

**Keywords:** carbon emissions, carbon footprint, environmental sustainability, environment change, maternal health

## Abstract

Environmental conditions closely influence maternal and newborn health, while healthcare practices in obstetrics and anesthesia can contribute to environmental impact through energy use, waste, and emissions. This editorial outlines practical strategies to enhance environmental sustainability in maternal healthcare. Improving energy efficiency through approaches such as modern lighting, optimized ventilation systems, and renewable energy sources can reduce resource consumption. Additional strategies include minimizing waste through reusable equipment, promoting telemedicine, and supporting low-intervention birthing options, where appropriate. Nurses and midwives play a key role by integrating sustainability into routine care and advocating for environmentally responsible practices, ensuring high-quality maternal care while contributing to a healthier, more resilient future for families and the planet.

## Editorial

The health of newborns is intricately connected to the well-being of mothers, with both being impacted by various environmental factors. Exposure to air pollutants during pregnancy, for example, can have adverse effects on both maternal and fetal health, influencing the long-term well-being of the newborn [1,2]. Within the healthcare sector, obstetrics and anesthesia practices impact the environment through energy use, waste production, and greenhouse gas emissions. Additionally, similar emissions originate from the use of anesthetic gases in other surgical fields, intensive care units, and emergency rooms, where nitrous oxide and volatile agents are frequently used. Anesthetic gases such as nitrous oxide, known for their potency as greenhouse gases, pose environmental implications [1,2]. It is imperative to enhance sustainability in healthcare by minimizing the environmental impact of obstetric procedures, optimizing anesthesia methods, reducing waste, and adopting energy-efficient approaches in healthcare facilities [1,2]. Addressing the carbon footprint of maternal and newborn health not only promotes environmental sustainability but also enhances overall public health outcomes. Sustainable healthcare strategies can result in improved health by minimizing exposure to harmful environmental pollutants. The intersection of healthcare and environmental sustainability underscores the need to lower the carbon footprint in healthcare services. As countries work towards achieving sustainable development goals, particularly SDG 3 (Good Health and Well-being) and SDG 13 (Climate Action), innovative solutions are crucial. This analysis delves into methods for reducing the carbon footprint in maternal healthcare, emphasizing energy efficiency, the adoption of sustainable practices, waste reduction, telemedicine utilization, and the development of pertinent policy frameworks [3]. The global health sector, including maternal healthcare, contributes substantially to greenhouse gas emissions, thereby worsening climate change. Key sources include healthcare facility operations, anesthesia and pharmaceuticals [1], transportation and infrastructure, and the interconnected effects of climate change on health and environmental justice [4]. The healthcare sector globally contributes around 4.6% of greenhouse gas emissions, stemming from various sources such as hospitals, clinics, pharmaceuticals, medical devices, and transportation linked to healthcare services [5]. Different estimates suggest a range of 1% to 5% for healthcare's contribution to global greenhouse gas emissions, reflecting diverse methodologies and regional contexts in emission assessments [5]. While specific data on greenhouse gas emissions from obstetric and newborn units within healthcare settings are limited, it is recognized that vulnerable groups such as newborns and expectant mothers may suffer notable health impacts from climate change, emphasizing the significance of integrating environmental sustainability in maternal healthcare practices.

Enhancing energy efficiency within healthcare facilities emerges as a notably efficacious approach for reducing the carbon footprint in maternal healthcare [6]. The utilization of energy-intensive apparatus and systems in medical centers and clinics plays a substantial role in carbon emissions. The integration of energy-efficient technologies such as LED (light-emitting diode) lighting, energy-efficient HVAC (heating, ventilation, and air conditioning) systems, and smart building management systems has the potential to result in a significant decrease in energy usage [7].

Embracing sustainable energy sources such as solar or wind power can further diminish the carbon footprint in healthcare environments. The installation of solar panels on the rooftops of hospitals presents a sustainable energy option, thereby decreasing reliance on fossil fuels [8]. Solar power could meet up to 30% of a hospital's energy needs in regions with abundant sunlight [9]. This transition not only diminishes emissions but also enhances energy security and generates long-term cost savings. The acquisition process represents another area where sustainability can be prioritized. Hospitals are encouraged to adopt eco-friendly procurement approaches by favoring suppliers that offer environmentally conscious products and sustainable production techniques. Opting for reusable medical equipment instead of disposable items can also result in waste minimization and a reduction in environmental impact [10].

The promotion of telemedicine and digital health solutions holds the potential to reduce the necessity for face-to-face consultations, thereby resulting in a decline in carbon emissions associated with the transportation of patients and staff. The integration of telemedicine can decrease carbon emissions by reducing transportation needs, particularly in rural and underserved medical areas. Telemedicine and other digital health solutions play a significant role in lowering the carbon footprint of healthcare delivery by minimizing patient and staff travel, improving healthcare accessibility, and reducing travel-related emissions [11]. This approach is particularly beneficial in prenatal and postnatal care, where regular check-ups can be efficiently conducted from a distance. Despite the advantages of remote prenatal and postnatal care in terms of convenience, accessibility, and continuity of care, challenges such as technology accessibility, limitations in physical examinations, and maintaining patient-provider relationships must be addressed. It is essential to strike a balance among these aspects when implementing remote care strategies effectively to enhance maternal health outcomes while mitigating potential disadvantages [12].

The implementation of sustainable practices, such as green surgeries in birthing procedures, plays a pivotal role in mitigating the carbon footprint of maternal healthcare services. Encouraging the practice of home births and the utilization of midwife-led birthing facilities, which typically consume fewer resources than hospital births, can prove to be beneficial for pregnancies categorized as low-risk [13].

Home births and midwife-led birthing facilities typically use fewer medical resources, such as pharmaceuticals, equipment, and hospital facilities, compared to hospital deliveries [14]. This can contribute to cost savings and reduce the environmental impact associated with medical waste and energy consumption in healthcare settings [15]. While home births and midwife-led facilities are suitable for low-risk pregnancies, pregnant individuals need to consult healthcare providers to assess their specific situation and to ensure that they receive appropriate prenatal care and have access to emergency services if needed [15].

These settings often use less energy-intensive equipment and produce less waste than hospital environments [16]. Strategies such as delayed cord clamping and immediate skin-to-skin contact not only enhance the health outcomes of newborns but also diminish the necessity for energy-intensive interventions. Encouraging the use of natural pain relief methods and non-pharmacological interventions can also reduce pharmaceutical waste and associated carbon emissions. Non-pharmacological pain relief methods, such as relaxation techniques, breathing exercises, massage, hydrotherapy, and acupuncture, are recognized in obstetric care for their effectiveness in managing labor pain and reducing the need for pharmaceutical interventions [17]. Reducing the use of pharmaceuticals in obstetric care lowers healthcare costs and decreases the carbon footprint associated with pharmaceutical production, transportation, and disposal [18]. Effective waste management is crucial in reducing the carbon footprint in maternal healthcare. Medical waste, especially from disposable products and packaging, has a significant impact on environmental contamination. Implementing effective waste segregation and recycling schemes can help alleviate this issue. Hospitals must collaborate with waste management entities to ensure the proper disposal and recycling of medical waste [19]. Improved inventory control and the avoidance of unnecessary prescriptions can also have a substantial impact.

Planetary health, defined as the interconnected well-being of human and natural systems, carries significant implications for the field of nursing and midwifery. Nurses and midwives, who serve on the front lines of healthcare provision, have pivotal roles in advancing maternal and child health and advocating for environmental stewardship [20]. A comprehensive understanding of how environmental elements such as air quality, climate variations, and the decline of biodiversity impact health empowers these professionals to champion policies and practices that safeguard both human well-being and the ecosystem. Incorporating the principles of planetary health into nursing and midwifery education equips practitioners with essential knowledge and skills to address emerging environmental health challenges and promote sustainable healthcare methods [21]. This holistic approach not only strengthens community resilience but also supports efforts to achieve health equity and sustainable development goals.

The initiation of policy measures and advocacy endeavors plays a critical role in driving systemic shifts towards sustainability within maternal healthcare. Governments and healthcare institutions must institute explicit policies and guidelines that prioritize sustainability [3]. Offering incentives to hospitals that meet energy efficiency goals or adopt eco-friendly practices, such as the use of eco-friendly construction materials, can foster widespread acceptance [22]. The integration of sustainability into medical education and training programs is essential to ensure that upcoming healthcare professionals possess the necessary knowledge and skills to promote and implement sustainable practices. The Lancet Countdown on Health and Climate Change (2021) emphasized the importance of incorporating climate change and sustainability into healthcare curricula to foster a culture of environmental stewardship among healthcare providers [5].

Collaboration among healthcare providers, policymakers, and communities is indispensable for sustainable maternal healthcare. This includes recognizing that maternal and newborn health are shaped not only by clinical care but also by environmental exposures, such as air pollution and the ecological impact of obstetric and anesthetic practices [1,2]. Engaging communities in sustainability initiatives, such as promoting the use of public transportation or carpooling for hospital visits, can help reduce the carbon footprint associated with patient transportation [23]. Healthcare facilities should also collaborate with local authorities and environmental organizations to develop and implement community-based sustainability programs. For example, programs encouraging the utilization of green spaces and physical activities can enhance maternal health outcomes while diminishing reliance on energy-intensive medical interventions [24].

Conclusion

The reduction of the carbon footprint in maternal healthcare requires a holistic approach that incorporates energy efficiency, sustainable practices, effective waste management, telehealth, and vigorous policy enforcement. Implementing these strategies will ensure that healthcare facilities can significantly reduce their environmental impact, thereby supporting global initiatives to address climate change while ensuring the health of mothers and their families. As we strive for a sustainable future, the healthcare sector must lead by example, demonstrating that environmental responsibility and the delivery of high-quality care can complement each other effectively.
